# Estimation of the number of inherited prion disease mutation carriers in the UK

**DOI:** 10.1038/s41431-022-01132-8

**Published:** 2022-06-27

**Authors:** Rosie Corbie, Tracy Campbell, Lee Darwent, Peter Rudge, John Collinge, Simon Mead

**Affiliations:** 1 National Prion Clinic, University College London (UCL) Hospitals NHS Foundation Trust, London, UK; 2 MRC Prion Unit at UCL, Institute of Prion Diseases, 33 Cleveland Street, London, W1W 7FF UK

**Keywords:** Neurodegeneration, Alzheimer's disease

## Abstract

Inherited prion diseases (IPD) are a set of rare neurodegenerative diseases that are always caused by mutation of the prion protein gene (*PRNP*). These are highly heterogeneous in clinical presentation and best described by the specific gene mutation, but traditionally include the canonical syndromes familial Creutzfeldt-Jakob disease, Gerstamann-Straussler-Scheinker syndrome, and fatal familial insomnia. In the UK, care of IPD patients and clinical *PRNP* sequencing have been carried out almost exclusively by the National Prion Clinic and affiliated laboratories since the disease gene was discovered in 1989. Using data obtained over 30 years (1990–2019), this study aimed to provide a greater understanding of the genetic prevalence of IPD using multiple complementary methods. A key source of bias in rare disorders is ascertainment, so we included an analysis based on capture-recapture techniques that may help to minimise ascertainment bias. 225 patients, with 21 different IPD mutations were identified, varying in frequency (with 8/21 mutations comprising over 90% observed cases), derived from 116 kindreds and 151 3-generation families. We estimated a total of 303 UK families (95% CI = 222, 384) segregate IPD mutations, 1091 (95% CI = 720, 1461) UK mutation carriers and a lifetime risk of approximately 1 in 60,000. Simpler methods of measuring prevalence based on extrapolation from the annual incidence of disease, and large scale genomic studies, result in similar estimates of prevalence. These estimates may be of value for planning preventive trials of therapeutics in IPD mutation carriers, prevention of prion disease transmission and provision of specialist services.

## Introduction

Prion diseases are rare untreatable neurodegenerative disorders of mammals caused by the propagation of prions, infectious agents comprised of polymers of misfolded forms of cellular prion protein (PrP^C^). They can occur sporadically, be acquired by dietary or iatrogenic exposures, or inherited (IPD), the latter constituting approximately 10–15% of the annual incidence of prion disease [1]. Over 50 different causal mutations, including missense variants, alteration of the number of octapeptide repeats, small indels elsewhere in the open reading frame, and truncations in the C-terminal region of the prion protein gene (*PRNP*), have been described which are predominantly inherited in an autosomal dominant manner. There is a spectrum of risk associated with mutations, whilst many mutations are known to be highly penetrant, some have low or uncertain penetrance [2, 3], others are best described as risk factors that only rarely cause a familial disease [4], and some missense variants are either benign or even protective against sporadic or acquired disease subtypes [5–8]. Individuals suffering from prion disease are also frequently misdiagnosed with other neurological conditions [1]. These features contribute to the difficulty in quantifying the occurrence of IPD, including its genetic prevalence meaning the proportion, or number of individuals in a population that are expected to be affected by a disease due to the possession of a pathogenic mutation [3, 9].

The UK has long had a well organised system for the assessment, diagnosis and care of patients with prion disease, with two national referral centres (in Edinburgh and London), each with a particular focus of expertise. The NHS National Prion Clinic in London has special responsibilities for IPD, where for the last 30 years and until recently, the affiliated MRC Prion Unit at UCL has been the sole provider of clinical genetic testing. This history presents a special opportunity to undertake a comprehensive analysis of the genetic prevalence of a disorder in one country. The information we learned might be useful for service provision and knowledge of prevalence of people carrying *PRNP* mutations provides part of the case for the commercial development and planning for early or preventive therapeutic interventions [10].

## Materials and methods

The data used in this analysis were obtained from NHS records held by the NPC between 1990 and 2019. Paper and electronic clinical files, patient letters, and findings from previous genealogical investigations using NPC data were used to construct family trees for each IPD case (the terms “kindreds”, “3-generation families” and “cases” are defined in Supplementary Material) [11, 12]. The capture-recapture method, originally developed to estimate the number of animals in a given habitat when counting them all was impractical, was recently adapted to estimate the number of families with a heritable disease [13]. Traditionally, this method involves capturing, marking and releasing animals in a ‘capture’ period, followed by capturing animals and counting those marked and unmarked in a ‘recapture’ period. The proportion of marked animals in the recaptured group should be equivalent to the proportion of the animals originally captured and marked in the total population. In our study, the number of different families with at least one IPD case diagnosed in an initial time period, the capture period, and the number of different families with at least one IPD case diagnosed within a second time period, the recapture period, were recorded. Families identified in the recapture period were classified as ‘marked’ if members of the same family were also identified in the capture period. The number of families ‘caught’, and the proportion of ‘marked’ families in the recapture period, were used to estimate the total family population size (formulae are detailed in Supplementary Material). This method was used to estimate the number of 3-generation families with IPD; the number of kindreds with IPD (ie known genealogies); and the number of mutations that cause IPD in the UK.

The capture-recapture method is underpinned by various assumptions which were tested and, where possible, mitigated against in this study (see Supplementary Materials). Importantly, there should be little migration in and out of the total population, which we measured, and can only be feasibly assumed if there is one provider of testing in a defined region. Notably, a further assumption is that capture and marking does not alter the probability of recapture. Plausibly, individuals may be more likely to be diagnosed in the recapture period if a family member was previously diagnosed in the capture period. We, therefore, tested the impact of altered ascertainment in diagnosed families. The average number of individuals suspected to harbour a *PRNP* mutation in an average family with IPD was calculated and used to estimate the genetic prevalence of IPD based on the capture-recapture results. This result was compared to simple estimates of genetic prevalence based on the incidence of IPD and large scale genome sequencing projects.

## Results

**Fig. 1:**
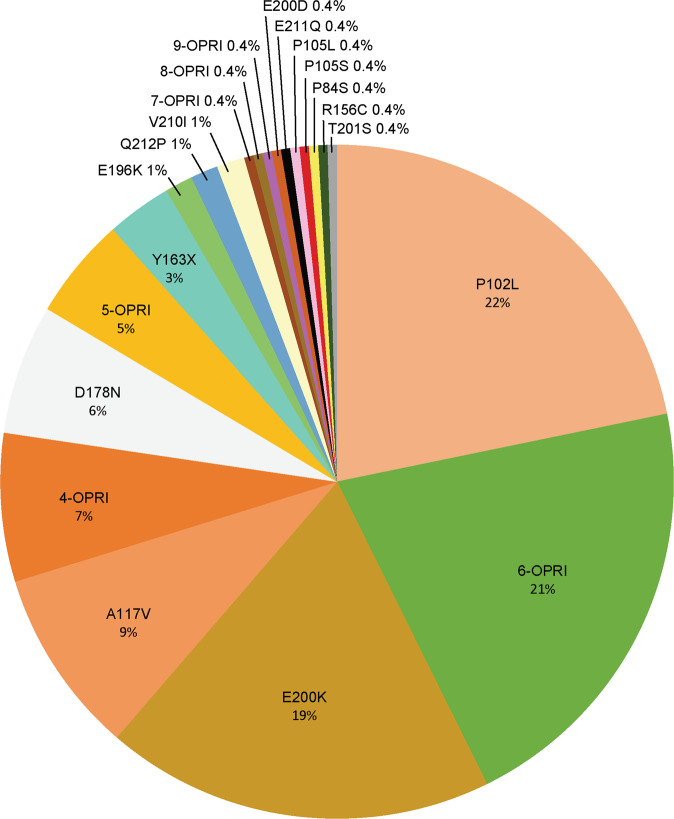
The Percentage of IPD Cases Caused by Each Mutation. Mutations are denoted in different colours and the percentage of patients with each mutation is shown.

225 patients, with 21 different IPD mutations were identified in the sample, varying in frequency (Fig. 1) and assumed to be fully penetrant and dominant for the purposes of the study, and derived from 116 kindreds and 151 3-generation families (Supplementary Table 1). Cumulatively, the 8 most common mutations account for over 90% of IPD cases, in order of frequency, p.P102L, 6-OPRI, E200K, A117V, 4-OPRI, D178N, 5-OPRI, and Y163X. We considered a capture period of 1990 to 2007 and recapture period of 2008 to 2019. These durations were chosen to approximate the numbers of cases in the capture and recapture periods as the annual diagnosis of IPD has increased over time. The capture-recapture method estimated 303 (95% CI 222–384) UK 3-generation IPD families (observed *n* = 151); 264 kindreds (observed *n* = 116); and 24 IPD mutations (observed *n* = 21) (Table 1).

**Table 1:** Capture-recapture analysis of the data obtained over the primary study period (Capture period: 1990–2007, Recapture period: 2008–2019).

	*m1*	*n2*	*m2*	Estimate	SE	Number Observed	Number of cases
3-generation families	83	93	25	302.7	41.3	151	225
Kinships	52	79	15	264.0	48.0	116	225
Mutations	14	17	10	23.6	2.3	21	225

The table shows the number of different 3-generation (3-gen) families, kinships and mutations identified in the capture period of 1990 to 2007 (m1) and in the recapture period of 2008 to 2019 (n2), along with the number of ‘marked’ families/mutations in the recapture period (m2). These values were used to generate an estimate for the number of 3-gen families, kinships and mutations (estimate) and the standard error of each estimate (SE). The overall number of families and individuals included in the analysis are given, along with the adjustments made to simulate the scenarios of increased ascertainment in marked families.

We tested the sensitivity of the method to various assumptions. Ascertainment bias might favour the diagnosis of a second patient with IPD in marked families. This type of bias increases our estimate of the number of 3-generation families by ~10 for each individual diagnosed with IPD in marked families that would have otherwise been missed (Supplementary Table 2). Using different shorter or equal length capture-recapture periods provided estimates of 3-generation families, kindreds, and mutations that were not meaningfully different to the results obtained from analysis above (Supplementary Tables 3 and  4).

The regional distribution of IPD patients was significantly different to the expected distribution of cases based on regional population averages, χ^2^(11, *n* = 198) = 98.8, *p* < 0.001. However, there was no significant difference between the observed and expected regional distribution of the number of patients with different IPD mutations, χ^2^(11, *n* = 74) = 5.8, *p* = 0.115. This suggests that high levels of geographic clustering of patients with the same mutation (perhaps due to founder effects) may be responsible for the seemingly inconsistent regional distribution of IPD cases. Low levels of migration were observed during this study: over the 30-year study period only 4 individual patients were found to be first-generation immigrants, we therefore did not adjust our analysis to take immigration and emigration into account.

### Genetic prevalence of IPD

The average age at death of patients is 55.4 years (*sd* = 12.0, *n* = 221). Based on the number of individuals in each generation and age-adjusted risks, we estimated that 4.04 individuals in a typical 3-generation IPD family will harbour a *PRNP* mutation (Supplementary Table 5). Based on the capture-recapture estimate of 303 (222–384) 3-generation pedigrees, this equates to 1297 individuals in total. Excluding patients that are now deceased gives the current UK IPD genetic prevalence of 1091 individuals, 1072 of whom are currently asymptomatic or undiagnosed (95% CI based on error in capture-recapture estimate, 720 to 1461).

Over the entire study period there was a positive correlation between number of deaths from IPD and calendar year (*r*_28_ = 2.576, *p* = 0.016). Annual incidence appears to plateau from 2004, and the range of diagnoses made per year decreases from this time. Indeed, no significant correlation was found between number of diagnoses and calendar year from 2004 to 2019 (*r*_14_ = −0.867, *p* = 0.401), when the average annual incidence was 9.3 (*sd* = 3.0, *n* = 148). Over this time period there were an average of 511,242 annual deaths in the UK, and 102.7 annual deaths from sporadic CJD, equating to a lifetime risk for an “average” person of 1/54,972 for IPD and 1/4978 for sporadic CJD. Note that the capture-recapture estimate of 1091 individuals comprises 1/61,485 of the 2020 ONS UK population estimate (67,081,000).

We looked at two large scale population genome databases for mutations reported that are known or likely to have penetrance ≥10%. UK Biobank reported a max 537496 alleles of *PRNP* including P102L (1), D1798N (1), E196K (1), E200K (1), V210I (1) and E196X (3) resulting in a prevalence of 1 in 33,594 (genebass.org); GnomAD v2.1.1 reported a max 282856 alleles of *PRNP* including P105S (1), E200K (1) and V210I (2) resulting in a prevalence of 1 in 35357 [3, 14].

## Discussion

In order to estimate the number of carriers of IPD mutations in the UK, we applied a technique developed in ecology that is used to measure population sizes when it is hard to count every individual. The capture-recapture method has been adapted to epidemiological situations, and particularly, in the estimation of the number of families with rare genetic conditions. We estimated the number of people who carry IPD mutations in the UK was 1091 based on the capture-recapture method (1/61,485 of current UK population), comparable to the 2004–2019 lifetime risk of death from IPD of 1/54,972.

The capture-recapture estimates obtained in this study may be biased due to deviations from the assumptions of the model. Repeating the capture-recapture analysis for short time periods produced similar results to those obtained using data from the entire study period. Immigration was minimal over the study period although, importantly, this will be one source of new IPD mutations and families in the UK (balanced by the loss by emigration). We tested the sensitivity of the method for ascertainment bias in favour of individuals with a family history of prion disease. Ascertainment biases have been implicated in false estimations of pathogenicity of IPD variants, and it would be reasonable to assume that an accurate diagnosis is more likely to be achieved when a family history is present.

The amount of information available regarding relatives varied between cases. Further genealogical investigations and genotyping could reveal distant relationships between cases that were not uncovered in this study. As a result, the estimated number of kinships may be slightly exaggerated, whilst the estimated number of 3-generation families is more reliable. Moreover, a fraction of patients who have IPD mutations are thought to have reduced penetrance [3]. Whilst the application of the capture-recapture method to families with genetic disease appears to us reasonable, as also noted by Ranola et al. [13], further investigations may establish better approaches to reduce bias and to validate the estimates generated.

Recent developments in the understanding of prion diseases suggest promising avenues towards therapeutics for IPD, and even the possibility of preventive treatment [10, 15]. Estimates that account for incomplete ascertainment reveal the full potential of such a treatment should awareness of possible treatments lead to improved recognition.

IPD diagnosis has implications for the potential iatrogenic transmission of prion diseases by surgery on high-risk CNS issues, and organ donation. Underdiagnosis can be largely attributed to misdiagnosis of other neurodegenerative conditions, partially due to the vast phenotypic variability and atypical manifestations of IPD [16]. Accordingly, the findings of this study suggest that *PRNP* sequencing should be considered when alternate diagnoses of neurodegenerative disorders are indefinite, although ascertainment going forwards will undoubtedly be impacted by the development of gene panel and clinical genome sequencing in the future [17–19]. Efficient diagnosis helps patients and relatives understand the course of the disease, the likelihood of treatment, and the risks of inheritance.

## Supplementary information

Supplementary Material

## Data Availability

From the corresponding author on reasonable request.
