# Supplementary material for: Estimation of the number of inherited prion disease mutation carriers in the UK
Source: Eur J Hum Genet. 2022 Jun 27;30(10):1167–70. doi: 10.1038/s41431-022-01132-8 (PMC9553982; doi:10.1038/s41431-022-01132-8)
Supplement: Supplementary file 1 — Supplementary Material [file 41431_2022_1132_MOESM1_ESM.docx]

**Supplementary Material**

| **Mutation** | **Number of Cases** | **Number of Kinships** | **Number of 3-generation families** |
| --- | --- | --- | --- |
| P102L | 49 | 19 | 30 |
| 6-OPRI | 47 | 3 | 19 |
| E200K | 42 | 37 | 38 |
| A117V | 20 | 4 | 9 |
| 4-OPRI | 16 | 12 | 13 |
| D178N | 14 | 12 | 12 |
| 5-OPRI | 11 | 8 | 8 |
| Y163X | 7 | 3 | 4 |
| E196K | 3 | 3 | 3 |
| Q212P | 3 | 3 | 3 |
| V210I | 3 | 2 | 2 |
| 7-OPRI | 1 | 1 | 1 |
| 8-OPRI | 1 | 1 | 1 |
| 9-OPRI | 1 | 1 | 1 |
| E200D | 1 | 1 | 1 |
| E211Q | 1 | 1 | 1 |
| P105L | 1 | 1 | 1 |
| P105S | 1 | 1 | 1 |
| P84S | 1 | 1 | 1 |
| R156C | 1 | 1 | 1 |
| T201S | 1 | 1 | 1 |
| TOTAL | 225 | 116 | 151 |

**Supplementary Table 1:** The number of cases, kinships, and 3-generation families identified in this study are shown for each mutation. Mutations are ordered by number of cases. Overall, 225 cases, 116 kinships and 151 3-generation families were ascertained.


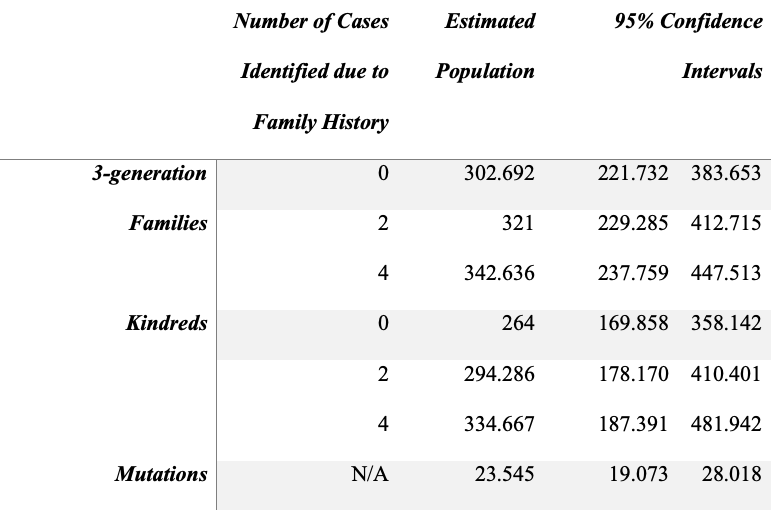


**Supplementary Table 2. Capture-recapture estimates of IPD 3-generation families, kindreds, and mutations.** The highlighted rows show results of an unadjusted analysis, estimating the number of 3-generation families with IPD; the number of kindreds with IPD; and the number of PRNP mutations. Lower and upper confidence intervals are given for each estimate. Adjusted estimates are also given for 3-generation families and kindreds, under the assumption that either an extra 2 or 4 cases were identified in the recapture phase because of increased ascertainment in the family due to the previous diagnosis.

|  | **Cases identified due to family history** | ***m1*** | ***n2*** | ***m2*** | **Estimate** | **SE** | **Number of families** | **Number of cases** |
| --- | --- | --- | --- | --- | --- | --- | --- | --- |
| **3-gen families** | 0 | 59 | 57 | 10 | 315.4 | 74.3 | 106 | 126 |
|  | 2 | 59 | 55 | 8 | 372.3 | 99.7 | 106 | 124 |
|  | 4 | 59 | 53 | 6 | 461.9 | 143.5 | 106 | 122 |
| **Kinships** | 0 | 48 | 48 | 8 | 265.8 | 68.9 | 88 | 126 |
|  | 2 | 48 | 46 | 6 | 328.0 | 99.3 | 88 | 124 |
|  | 4 | 48 | 44 | 4 | 440.0 | 160.8 | 88 | 122 |
| **Mutations** | N/A | 12 | 14 | 9 | 18.5 | 1.6 | 17 | 126 |

**Supplementary Table 3. Capture-Recapture Analysis of Data Obtained Between 2004 and 2019 (Capture Period: 2004-2012, Recapture Period: 2013-2019).** The table shows the number of different 3-generation (3-gen) families, kinships and mutations identified in the capture period of 2004 to 2012 (m1) and in the recapture period of 2013 to 2019 (n2), along with the number of ‘marked’ families/mutations in the recapture period (m2). These values were used to generate an estimate for the number of 3-gen families, kinships and mutations (estimate) and the standard error of each estimate (SE). The overall number of families and individuals included in the analysis are given, along with the adjustments made to simulate increased ascertainment due to the previous diagnosis in the family.

|  | **Cases identified due to family history** | ***m1*** | ***n2*** | ***m2*** | **Estimate** | **SE** | **Number of families** | **Number of cases** |
| --- | --- | --- | --- | --- | --- | --- | --- | --- |
| **3-gen families** | 0 | 46 | 40 | 4 | 384.4 | 139.4 | 82 | 91 |
| **Kinships** | 0 | 40 | 35 | 5 | 245.0 | 78.4 | 70 | 91 |
| **Mutations** | N/A | 13 | 11 | 7 | 20.0 | 2.6 | 17 | 91 |

**Supplementary Table 4. Capture-Recapture Analysis of Data Obtained Between 2010 and 2019 (Capture Period: 2010-2014, Recapture Period: 2015-2019).** The table shows the number of different 3-generation (3-gen) families, kinships and mutations identified in the capture period of 2010 to 2014 (m1) and in the recapture period of 2015 to 2019 (n2), along with the number of ‘marked’ families/mutations in the recapture period (m2). These values were used to generate an estimate for the number of 3-gen families, kinships and mutations (estimate) and the standard error of each estimate (SE).

| **Group of Relatives** | **Number of individuals** | **Risk** | **Age** | **Age Adjusted Risk** | **Genetic Prevalence** |
| --- | --- | --- | --- | --- | --- |
| Generation II (FDR) | 1 | 1 | 96.0 | 0.001 | 0.001 |
| Generation II (SDR) | 1 | 0.5 | 96.0 | 0.000 | 0.000 |
| Generation III (FDR) | 2.279 | 0.5 | 68.0 | 0.072 | 0.164 |
| Generation III (TDR) | 2.12 | 0.25 | 68.0 | 0.036 | 0.076 |
| Generation III (Proband) | 1 | n/a | n/a | n/a | 1 |
| Generation IV (FDR) | 2.090 | 0.5 | 41.0 | 0.409 | 0.855 |
| Generation IV (SDR) | 4.603 | 0.25 | 41.0 | 0.205 | 0.941 |
| Generation V (SDR) | 4.013 | 0.25 | 11.7 | 0.250 | 1.002 |
| TOTAL |  | | | | 4.040 |

**Supplementary Table 5. Age-adjusted probabilities of harbouring a PRNP mutation for each group of relatives in an average IPD family.** Individuals in an average IPD family (up to those at 25% risk) are grouped by generation and degree of relation to the proband, including first-degree relatives (FDR), second-degree relatives (SDR) and third-degree relatives (TDR). The average number of individuals in each group, along with their theoretical risk of harbouring a *PRNP* mutation (Risk) is given. According to the average age of each group (Age), the risk of harbouring a PRNP mutation for an average individual in each group was adjusted (Age-Adjusted Risk). Genetic Prevalence was subsequently deduced for each group and the average IPD family as a whole.

**Supplementary methods**

*Data*

All suspected IPD cases undergo sequencing of the complete *PRNP* open reading frame, and relatives may choose to undergo predictive testing. Overall, 225 positive diagnostic tests and 214 predictive tests, comprising 127 predictive negative results and 87 predictive positive results, have been performed between 1990 and 2019. Presymptomatic testing is only offered to relatives of individuals diagnosed with IPD, therefore including individuals who have undergone this form of testing would bias results. Moreover, only 13 individuals that previously received predictive testing have become symptomatic since 1990. As a result, only symptomatic individuals who received diagnostic testing were used in this analysis. Statistical analyses on this data were performed using Excel and RStudio. Concluding statistics referring to numbers of individuals, families, or mutations were rounded to the nearest integer for coherence.

*Capture-Recapture Analysis*

Traditionally, the capture-recapture method involves capturing, marking and releasing animals in a ‘capture’ period, followed by capturing and counting marked animals in a ‘recapture’ period^1^. The proportion of marked animals in the recapture period should be equivalent to the proportion of marked animals in the whole population, which is the principle used to estimate the total population. This method has been applied to families. Our study involved counting the number of different families with at least one IPD case diagnosed in an initial time period (m1), the capture period. The number of different families with at least one IPD case diagnosed within a second time period (n2), the recapture period, were subsequently obtained. Families identified in the recapture period were classified as ‘marked’ (m2) if members of the same family were also identified in the capture period. This procedure was applied to 3-generation families, kindreds, and mutations. The number of IPD families or mutations (*N*) is estimated using the equation n = [{(m1+1)*(n2+1)}/(m2+l)]-1^2^ which is adjusted for small sample sizes. 95% confidence intervals were calculated using the adapted formula CI = N±1.96*SE, where standard error is calculated using the formula SE = sqrt{[(m1+l)(n2+l)(m1-m2)(n2-m2)]/(m2+l)2(m2+2)}^2^.

*Descriptions of capture-recapture estimated values*

The capture-recapture method was used to estimate the number of 3-generation pedigrees with IPD; the number of kindred with IPD; and the number of mutations that cause IPD in the UK. 3-generation pedigrees were defined as relatives of an IPD patient extending out to first cousins. In the rare event that multiple closely related patients were found in a multi-generation pedigree, the pedigree was split such that no 3-generation pedigree contained cases that were more distantly related than first cousins, and minimal overlapping of pedigrees. Kindreds were specified as patients with any known relationship, which includes close and distant relatives even if this had been defined at the NPC by genealogical research. The number of mutations simply describes the count of pathogenic *PRNP* mutations in the population. For clarity, the use of the term ‘family’ in this study refers more generally to a group of related individuals which includes both 3-generation pedigrees and kindreds.

*Identifying IPD families*

Patient letters and any related documents were searched for the mention of relatives. Upon diagnosis, most clinicians would take note of any relatives as they may wish to have predictive testing, aiding this process. Multi-generation pedigrees constructed in previous studies through genotyping and genealogical research using NPC data were also used to ascertain information on family members^6,7^. These data were compiled and used to assign every IPD case to a 3-generation pedigree, kindred, and mutation group.

*Assumptions and modifications of the capture-recapture method:*

The capture-recapture method is underpinned by various assumptions which are tested and, where possible, mitigated against in this study. As fewer individuals were ‘caught’ when genetic testing for IPD was first introduced, presumably because of clinician awareness and access issues, the timeframes used in this analysis were adjusted such that roughly equal numbers of diagnoses were made in each period. Hence, the primary capture period was defined as 1990 to 2007 and the primary recapture period as 2008 to 2019. Two shorter, more recent periods, over which diagnoses were more constant, were also assessed to test the variability of estimates based on the definition of the periods of capture and recapture. Pearson’s chi-squared tests were used to determine whether an individual’s location or proximity to the NPC affected the probability of ‘capture’. An individual being diagnosed with IPD in the capture period may increase the chances that a family member is subsequently diagnosed in the recapture period, perhaps because of increased awareness of the disease in the family. The capture-recapture calculation was therefore modified by assuming that a given number of individuals (2 or 4), with one other family member identified in the capture period, would not have otherwise been diagnosed. The number of families identified in the recapture period, and the corresponding ‘marked’ value, were reduced in these scenarios which inflated the estimate of mutation carriers in the UK. We also tested the effects of altering the length of the capture and recapture time frames and making note of migrants assessed the impact of the UK being an open population.

### *Estimating IPD Genetic Prevalence from Capture-Recapture Findings:*

The average family tree of a proband, extending to relatives at a theoretical 25% risk of IPD, was constructed. The average number of first-degree relatives and age of individuals in the same generation as the proband were estimated using patient averages. The age of individuals in different generations and number of individuals in each generation were subsequently estimated using population averages^3,4^. Individuals were grouped by generation and degree of relatedness to the proband. The probability that an individual harboured a *PRNP* mutation was calculated for each group of relatives based on an assumed autosomal dominant inheritance pattern and amended according to approximate age. Multiplying probability of harbouring a *PRNP* mutation by the average number of individuals in each group returned the average number of individuals suspected to harbour a *PRNP* mutation in an average IPD family. To generate an estimate of genetic prevalence, this average was further multiplied by the estimated number of 3-generation pedigrees.

## *Estimating Genetic Prevalence of IPD Based Upon Incidence:*

The average annual incidence of IPD was deduced from the years whereby the number of positive IPD diagnostic tests appeared to plateau. Pearson’s product-moment correlation coefficients were calculated to determine whether any correlations in this data were significant. Multiplying incidence by the average age of diagnosis provided another estimate of genetic prevalence.

**Supplementary References**

1 Stephen C. Capture-Recapture Methods in Epidemiological Studies. *Infect Control Hosp Epidemiol* 1996; **17**: 262–6.

2 Chapman D. *Some properties of the hypergeometric distribution with applications to zoological sample censuses*. University of California Press: Berkeley, 1951.

3 Office for National Statistics. Births by parents’ characteristics. 2020. https://www.ons.gov.uk/peoplepopulationandcommunity/birthsdeathsandmarriages/livebirths/datasets/birthsbyparentscharacteristics. Accessed 14 Apr 2020.

4 Office for National Statistics. Childbearing for women born in different years. 2020. https://www.ons.gov.uk/peoplepopulationandcommunity/birthsdeathsandmarriages/conceptionandfertilityrates/datasets/childbearingforwomenbornindifferentyearsreferencetable. Accessed 11 Apr 2020.
